# Surface Chemistry and Molecular Dynamics of Epoxy Resin: Insights from Analysis During Curing and Post-Curing Processes

**DOI:** 10.3390/polym17081094

**Published:** 2025-04-18

**Authors:** Bogdan-Marian Tofanica, Elena Ungureanu, Firas Awaja

**Affiliations:** 1 “Ion Ionescu de la Brad” Iasi University of Life Sciences, 3 Mihail Sadoveanu Alley, 700490 Iasi, Romania; bogdan.tofanica@iuls.ro; 2 School of Medicine, University of Galway, H91 TK33 Galway, Ireland; firas.awaja@nuigalway.ie

**Keywords:** epoxy resin, curing process, surface reactions, cross-linking reactions, molecular structure, principal components regression

## Abstract

The surface chemistry of epoxy resin and its composites is critical for their long-term performance across various applications. In this study, we investigate the main reactions occurring on the surface of DEGBA/DEGBF epoxy resin following curing, post-curing, and thermal post-curing processes using Time-of-Flight Secondary Ion Mass Spectrometry (ToF-SIMS). ToF-SIMS analysis elucidated molecular details, including curing and cross-linking progression, cross-link characteristics, cured resin structure, residual unreacted hardener, cross-linking density, and reaction pathways. Principal Components Regression analysis (PCR) was applied to distinguish between cured and post-cured samples, focusing on specific ions indicative of the curing process. The completion of curing was associated with ions such as C_14_H_7_O^+^, CHO^+^, CH_3_O^+^, and C_21_H_24_O_4_^+^, while unreacted hardener was indicated by C_21_H_24_O_4_^+^ ions. Cross-linking density and the intensities of aliphatic hydrocarbons were crucial in differentiating curing stages. Calibration ensured that all ion intensities totaled to one, and specific ions were tracked to monitor the states from uncured to post-cured. Negative spectra provided insights into the consumption of hardener molecules during curing and post-curing. The results demonstrated that post-curing enhances the properties of epoxy resin by promoting further cross-linking, reducing residual unreacted groups, and forming a more extensive covalent network. This results in improved mechanical and thermal stability. The molecular changes observed through ToF-SIMS data effectively distinguish between curing and post-curing reactions, contributing to a better understanding and optimization of epoxy resin properties for various applications.

## 1. Introduction

Epoxy resins have wide-ranging applications as adhesives, coatings, and composites in industries such as automotive and aerospace [1,2,3]. Moreover, ecological biocide systems utilizing unmodified or epoxidized lignins, furan resin, and copper are emerging as promising bioprotective solutions. Applications of epoxidated lignins, in particular, are being explored for the bioprotection of lignocellulosic materials, further expanding the versatility and sustainability of epoxy-based technologies [4,5].

The curing and cross-linking reaction of epoxy resin is a complex process that involves multiple reaction pathways [6]. Despite their significance, the molecular mechanisms of these reaction pathways remain incompletely understood [7]. The chemical structure of epoxy resins is a crucial factor in determining their application and performance, and is influenced by the initial components and curing reaction settings during manufacturing [3].

The cross-linked epoxy resin is typically obtained through the curing reaction of a bi-functional epoxide such as Bisphenol A diglycidyl ether (CAS Number 1675-54-3, commonly abbreviated DGEBA) or Bisphenol F diglycidyl ether CAS Number 2095-03-6, commonly abbreviated DGEBF) with a multifunctional cross-linker/curing agent, otherwise known as a hardener. The most commonly used cross-linking agent is the amine-type, which is tetrafunctional and has hydrogen atoms on the sides of the nitrogen atoms serving as reacting units. The nitrogen atoms react with the epoxy group on the resin, resulting in the formation of a new carbon bond and a hydroxyl functional group [3]. The tetra functionality of the cross-linking agent enables it to react with four epoxide groups, leading to a cross-linked structure. Ideally, the final product is a fully cross-linked network or a single giant molecule. Nevertheless, the incomplete conversion of the curing reaction, combined with the inherent complexity of the reaction pathways, leads to the simultaneous presence of both linear and branched polymer architectures, ultimately influencing the final product properties [3].

A detailed understanding of the molecular architecture of these polymer chains is essential for controlling their formation and optimizing their distribution within the final cured network. Real-time monitoring of the epoxy resin curing process offers greater precision in tailoring the final material specifications. Moreover, in-depth molecular characterization of the cured epoxy structure provides critical insights into structure–property relationships and contributes to improved resistance against environmental degradation mechanisms.

A wide range of both off-line and on-line techniques have been developed for monitoring the progress of curing reactions [3,8,9,10,11,12,13]. Off-line methods such as titration, specific gravity measurements, and differential scanning calorimetry (DSC) are commonly employed by extracting samples at specific times during the curing reaction for subsequent analysis [7,14]. Among these, DSC is particularly valuable for elucidating reaction kinetics, including parameters as reaction activation energy, frequency factor, and reaction order, as well as for kinetics modeling [6,7,14,15,16,17,18,19].

Many researchers have attempted to elucidate the reaction chemistry and physics of the DGEBA/diamine systems [7,16,20]. The curing reaction of DGEBA with different imide-amine and diamine hardener concentrations using DSC has been previously described [6,15]. Pichaude et al. [16] studied the influences of hydroxyl content on the curing kinetics of DGEBA with IPD as a hardener, while Lopez-Quintela et al. [20] investigated the cis/trans reactivity of the DGEBA epoxy/diamine mixtures. The morphology of the cured epoxy resin, which directly governs its mechanical, thermal, and chemical properties, is strongly influenced by both the intrinsic nature and the kinetics of the cross-linking reaction between the resin and the curing agent [19]. Numerous studies have demonstrated that the structure and functionality of the hardener, along with its concentration, play a critical role in determining the extent of network formation and the resulting material performance [15].

In contrast, on-line monitoring techniques employ embedded sensors that are integrated directly into the epoxy matrix during the curing process, enabling real-time transmission of data to external systems for continuous tracking of the reaction kinetics and conversion progress. One such method is low-frequency dielectrometry, which measures permanent dipoles within the structure and the mobility of impurities in the resin matrix. These quantities correlate with resin viscosity and mechanical rigidity, which in turn relate to reaction conversion [8,9]. Infrared and Raman spectroscopy have been used in combination with optical fibers implanted in the curing resin to transmit spectral information to an outside monitoring system [10,11,12,13].

Beyond the primary curing reaction, the subsequent post-curing and thermal post-curing stages are critical for achieving complete cross-linking and ensuring enhanced mechanical strength, thermal stability, and environmental durability of epoxy resin systems. These additional curing phases provide a deeper insight into the evolution of network structures and allow for improved optimization of processing conditions and final material performance.

While conventional techniques such as differential scanning calorimetry (DSC), titration, and off-line gravimetric measurements offer valuable insights into the kinetics and thermal properties of epoxy resin curing, they often fall short in providing real-time, molecular-level details of the dynamic curing process. These limitations hinder a comprehensive understanding of the intricate reaction pathways and transient intermediates that govern network formation.

Our study seeks to bridge this gap by employing time-of-flight secondary ion mass spectrometry (ToF-SIMS), which offers the high-resolution, real-time analytical capability needed to elucidate these molecular transformations. In this work, the progress of the curing reaction of DGEBA and DGEBF epoxy resin blend reacted with isophorone diamine (IPD) based hardener is followed using ToF-SIMS. Molecular changes are revealed as a function of time for the epoxy resin and IPD hardener curing reaction.

ToF-SIMS is a powerful technique that has the potential to provide detailed information about molecular changes as the curing reaction progresses. Unlike other monitoring methods, ToF-SIMS offers exceptional surface sensitivity and chemical specificity, making it particularly well-suited for detecting subtle changes in molecular composition and structure during the curing of epoxy systems. In this work, we exploit this capability to monitor variations in the intensities of ion fragments derived from the resin, hardener, and their reaction products. These spectral changes are evaluated using both univariate and multivariate statistical methods to uncover meaningful trends in the curing process.

The utility of ToF-SIMS in studying thermosetting resins has been demonstrated in prior studies. For instance, Coullerez et al. [21] employed ToF-SIMS to compare the chemical structure of melamine formaldehyde resin in its uncured and fully cured states, revealing additional information about the chemical structure of the resin. Similarly, Rattana et al. [22] utilized ToF-SIMS to examine the adsorption behavior and thermodynamics of DGEBA epoxy resin molecules on silane-treated aluminum substrates, providing detailed information on monolayer coverage and interfacial chemistry.

Due to the inherent complexity of ToF-SIMS spectra, advanced data analysis techniques such as Principal Component Analysis (PCA) are often required to extract meaningful information. PCA has been widely applied in the context of polymer and surface chemistry to facilitate the interpretation of multicomponent spectra [23,24]. Prior to PCA, appropriate data preprocessing, such as scaling and normalization, is essential to ensure that observed variations reflect true chemical differences rather than instrumental artifacts like fluctuation in secondary ion yield or detector sensitivity [23,24].

## 2. Materials and Methods

In this study, we utilized KINETIX R246TX epoxy resin (ATL Composites, Molendinar, Australia) a commercial formulation primarily composed of diglycidyl ether of bisphenol A (DGEBA), blended with diglycidyl ether of bisphenol F (DGEBF) and an aliphatic glycidyl ether-functional reactive diluent. As the curing agent, we employed KINETIX H126 (ATL Composites, Australia), specifically designed for room-temperature curing. The hardener formulation is based predominantly on isophorone diamine (IPD), a cycloaliphatic diamine known for promoting rapid cross-linking and imparting good mechanical performance. For the ToF-SIMS comparison, samples that had undergone various treatments were examined: the resin alone; a resin-to-hardener mixture at a 4:1 ratio (HR4); curing for 4 h at 22 °C (C1); curing for 24 h at 22 °C (C2); post-curing for 4 h at 100 °C (TP100); and post-curing for 4 h at 120 °C (TP120).

**Figure 1:**
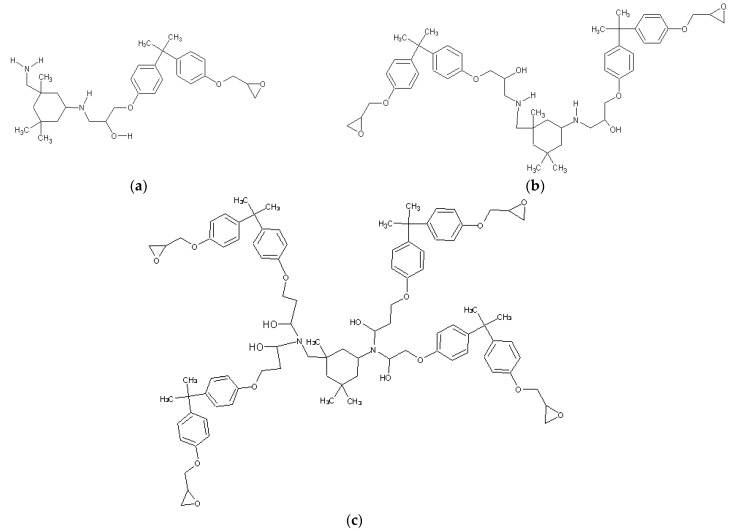
Structures of the epoxy resin curing reactions of DGEBA/IPD molecules of (**a**) blocking, (**b**) linear coupling and (**c**) Branching formations.

A schematic of the cross-linking structure formed between DGEBA and IPD is presented in Figure 1. The curing reaction proceeds via nucleophilic attack of the amine groups of IPD on the epoxide rings, resulting in β-hydroxy ether linkages. This reaction leads to the formation of a densely cross-linked thermoset polymer network. The representation serves as a reference for interpreting the evolution of chemical species and cross-linking density discussed in subsequent sections.

### 2.1. ToF-SIMS

A ToF-SIMS IV instrument (Ion-TOF GmbH, Münster, Germany) equipped with a reflectron time-of-flight mass analyzer, a Bi^3+^ ion gun (25 keV), and a pulsed electron flood source for charge compensation was used to perform the analyses. The primary pulsed ion beam current was 1.1 pA, and the primary ion dose density was below the dynamic SIMS limit of 10^13^ ions/cm^−2^. Positive spectra with high mass resolution (>7500 at m/z 29) were acquired from a 100 µm × 100 µm fresh spot at each time interval, with a 100 µs cycle time.

The resin and hardener were mixed at a 4:1 ratio, per the manufacturer’s recommendation, for 10 min. The prepared resin–hardener mixture was spin-coated onto a silicon wafer substrate to form a uniform thin film. Immediately following deposition, the sample was introduced into the ToF-SIMS load-lock chamber for initial evacuation. Upon reaching a sufficiently low pressure, the sample was transferred into the main analysis chamber, where high-vacuum conditions—better than 2 × 10^−8^ torr—were maintained to ensure optimal surface sensitivity and signal stability during analysis. Positive ion spectra were acquired 40 min after the resin and hardener mixture was applied to the Si wafer. The sample was then heated to 120 °C and kept at that temperature for 3 h. Finally, five positive spectra were acquired.

Vacuum exposure during ToF-SIMS can lead to minor surface composition changes, particularly in low molecular weight or volatile species. However, prior studies [23,24] indicate that the core structure remains stable during analysis. Nevertheless, caution is applied in interpreting results for uncured or partially cured samples.

### 2.2. Data Analysis

Initially, peaks for data analysis were chosen based on reference libraries and previous assignments in the literature for DGEBA, DGEBF, and IPD molecules. Then, the library and the exact mass calculator tool in the Ionspec software (Ion-TOF GmbH, Münster. Germany), which is part of the SurfaceLab suite designed for TOF-SIMS data acquisition and analysis, were employed to detect potential contamination, including hydrocarbon-related peaks, as well as to detect previously unreported fragment ions arising from the resin and hardener components that are not documented in the existing literature. All significant peaks above baseline in the m/z range of 0–300 were chosen, as were significant peak intensities that could be resolved and related to the DGEBA, DGEBF, and IPD molecules in the range of m/z 300–650. The mass spectra were calibrated using a series of hydrocarbon (CxHy) peaks up to m/z = 105.

The data were then grouped into a matrix, with columns normalized to the total intensity and mean-centered before being used for Principal Component Analysis (PCA). PCA is a powerful multivariate statistical tool widely employed to facilitate the interpretation of complex, high-dimensional datasets such as those produced by ToF-SIMS. By transforming the original variables into a reduced set of orthogonal principal components, PCA captures the dominant sources of variance within the data. These components are derived through singular value decomposition of the data, allowing the most significant patterns, such as chemical trends or material changes, to be distinguished from background noise. This dimensionality reduction not only simplifies visualization and analysis but also aids in the identification of key molecular fragments or spectral features relevant to the curing process.

PCA was conducted using a combination of custom-developed scripts and the statistical functions provided in the Stats package (v2.5.1) within the R environment for statistical computing and graphical analysis (R version 4.3.0). The implementation follows the covariance-based algorithm described in detail by Martens and Næs [25]. Specifically, the covariance matrix was computed from the mean-centered data matrix, and its eigenvalues were extracted and sorted in descending order to reflect the proportion of variance captured by each corresponding eigenvector. These eigenvectors, calculated from the covariance matrix and eigenvalues, represent the new orthogonal basis vectors, i.e., the principal components that capture the structure of variance within the dataset. The sorted eigenvalues provide a quantitative measure of the variance each component explains, allowing us to distinguish the most informative dimensions.

Typically, the first few principal components account for the majority of the systematic variance, while subsequent components capture residual or noise-level information. The final set of principal components was then calculated by projecting the mean-centered original data matrix onto the eigenvector space.

PCA is typically discussed in terms of scores and loadings plots. The scores plots are the principal components plotted against each other or against the main variables in the dataset, while the loadings represent the relative contribution of each mass peak in the peak list to the principal components. The loadings plots are the eigenvectors plotted against the spectra mass values.

## 3. Results and Discussion

Comprehending the progression of curing and cross-linking, cross-link nature and extent, cured resin structure, residual unreacted hardener, cross-linking density, and reaction pathways is critical for optimizing epoxy resin applications. These factors critically influence the mechanical, thermal, and chemical properties of the final product, which, in turn, dictate its suitability for specific applications.

The progression of curing and cross-linking reactions determines the development of the polymer network structure within the epoxy resin. By monitoring these reactions, one can ensure that the curing process reaches completion, resulting in a material with optimal mechanical properties and durability. The nature and degree of cross-links are essential as they define the rigidity and strength of the cured resin. A well-cross-linked resin exhibits superior resistance to mechanical stress and environmental degradation, making it ideal for high-performance applications such as aerospace, automotive, and electronics.

The structure of the cured resin is another critical aspect, as it affects the material’s performance characteristics. A homogeneous and well-structured resin ensures uniform mechanical properties, while an improperly cured resin may exhibit weak spots and vulnerabilities. Additionally, the presence of remaining and unreacted hardener can lead to incomplete curing, compromising the material’s integrity and potentially causing failure in its application. Therefore, it is essential to understand and control the amount of unreacted hardener to achieve a fully cured and stable resin.

Cross-linking density is a measure of the number of cross-links per unit volume in the cured resin. This parameter directly influences the material’s thermal and mechanical properties. A high cross-linking density typically results in a more rigid and heat-resistant material, whereas a lower density may lead to a more flexible but less durable product. Understanding the curing reaction pathways is equally important as it provides insights into the chemical processes involved in the formation of the epoxy network. This knowledge enables the optimization of curing conditions and the selection of appropriate hardeners and additives to achieve desired properties in the final product.

Time-of-Flight Secondary Ion Mass Spectrometry (ToF-SIMS) emerges as a powerful tool for evaluating these critical aspects of epoxy resins. ToF-SIMS allows for detailed surface analysis and characterization of the resin at the molecular level. It provides valuable information on the chemical composition, distribution of elements, and identification of molecular fragments within the resin. This technique is particularly useful for detecting the presence of unreacted hardeners and monitoring the progression of curing reactions.

Moreover, ToF-SIMS can map the spatial distribution of cross-links and other chemical species within the cured resin, providing insights into the uniformity and completeness of the curing process. This capability is crucial for identifying potential defects or inconsistencies in the material, which could compromise its performance. The high sensitivity and resolution of ToF-SIMS make it an indispensable tool for ensuring the quality and reliability of epoxy resins in demanding applications.

A comprehensive understanding of curing and cross-linking reactions, cross-linking density, and the chemical structure of epoxy resins is essential for optimizing their performance and reliability. ToF-SIMS plays a vital role in this evaluation process, offering precise and detailed analysis that helps in the development and quality control of high-performance epoxy resins.

### 3.1. Completion of Curing Reaction

Epoxy resins are thermosetting polymers cured by reacting epoxide functional groups with a curing agent, typically a hardener. The curing reaction is exothermic, forming covalent bonds between the epoxide groups and the curing agent. This process occurs in two stages: an initial fast reaction involving the opening of the epoxy ring and formation of reactive intermediates, followed by a slower reaction that crosslinks these intermediates to form a three-dimensional network. Completing the curing reactions in epoxy resins is crucial to achieving the material’s full mechanical and chemical properties. From a molecular chemistry perspective, completing the curing reactions involves consuming all reactive epoxide groups and forming a crosslinked network of covalent bonds. This network structure endows the epoxy resin with its unique combination of mechanical, thermal, and chemical properties, such as high strength, rigidity, and resistance to heat and chemicals.

The curing reaction of epoxy resins is complex and depends on various factors, including the type and concentration of the hardener, curing temperature, and curing time. Reaction kinetics may vary with different curing systems, affecting the degree of curing and the resulting properties of the epoxy resin. Therefore, optimizing the curing conditions is essential to achieve the desired properties for a particular application.

**Figure 2:**
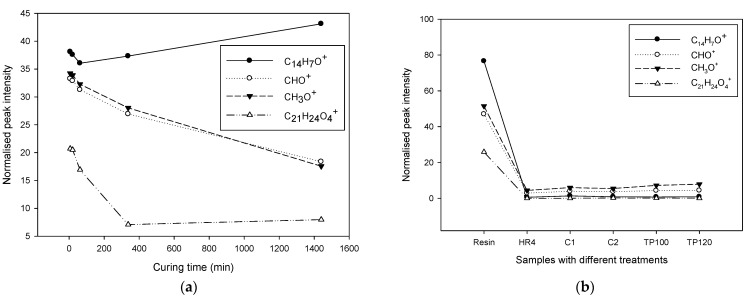
This figure shows ToF-SIMS normalised peak intensity of selected main molecules with (**a**) curing time, and (**b**) samples with different treatments.

**Figure 3:**
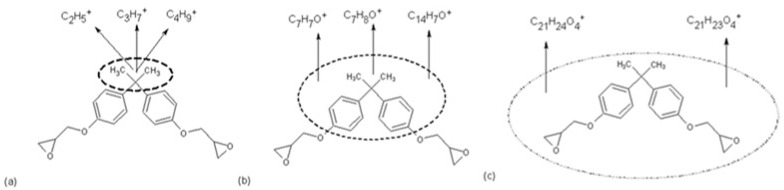
Model fragmentation of the primary ion and examples of the resulting secondary ions on DGEBA molecule for the (**a**) violent fragmentation region, (**b**) low energy fragmentation region, (**c**) structural fragmentation region.

Figure 2 illustrates the Time-of-Flight Secondary Ion Mass Spectrometry normalized peak intensity of selected main molecules, providing insights into the curing dynamics of the epoxy resin. Complementarily, Figure 3 presents a model fragmentation of the primary ion and examples of the resulting secondary ions on the DGEBA molecule, highlighting different fragmentation regions.

The data are presented in two subfigures:

- Figure 2a: Normalized Peak Intensity with Curing Time—this subfigure depicts the normalized peak intensity of the main resin molecules as a function of curing time. As curing progresses, the peak intensities of C_21_H_24_O_4_^+^ and other key ions (e.g., C_14_H_7_O^+^, CHO^+^, CH_3_O^+^) decrease, reflecting their reduced concentrations. The decrease in peak intensity past the recommended 380 min suggests that the majority of the curing reaction occurs within this timeframe. However, the continuation of curing and post-curing reactions up to 1400 min, as evidenced by the ongoing reduction in peak intensities of these molecules, indicates that some reactions and molecular changes continue beyond the initial curing period. C_21_H_24_O_4_ is the molecular formula for Bisphenol A diglycidyl ether (BADGE), which is a common epoxy resin. It is formed by the reaction of bisphenol A (BPA) with epichlorohydrin, and is widely used in the manufacture of epoxy resins due to its excellent mechanical properties and chemical resistance. The molecular formula C_14_H_7_O corresponds to anthracene-9,10-quinone, also known as anthraquinone. Anthraquinone is a derivative of anthracene, characterized by the presence of two ketone groups (carbonyl groups) at the 9 and 10 positions of the anthracene molecule [26,27].
- Figure 2b: Normalized Peak Intensity with Different Treatments—this subfigure compares the normalized peak intensities of the selected main molecules across samples subjected to different treatment conditions. Regardless of the specific treatment type, the trend shows a consistent decrease in the concentration of these molecules. This indicates that the curing reactions follow a similar pathway across different treatments, leading to a reduction in the presence of the original resin molecules and intermediates. The convergence of peak intensities to nearly undetectable levels across all treatments suggests that the curing process effectively consumes the reactive molecules and promotes the formation of the final crosslinked network, albeit with variations in the rate or efficiency of this process depending on the treatment specifics.

These ToF-SIMS results collectively demonstrate the efficacy and progression of the curing reactions in epoxy resins. By monitoring the normalized peak intensities of selected molecules, we can better understand how different curing times and treatment conditions affect the consumption of reactants and the development of the final polymer network. This detailed molecular-level analysis aids in optimizing the curing process to ensure that the epoxy resin achieves its desired mechanical, thermal, and chemical properties for various applications.

The resin ion C_21_H_24_O_4_^+^ is unlikely to be detected after the coupling reaction due to the specific fragmentation required, which is not possible when it is attached to other molecules. However, it has a better chance of being detected if the molecule is unreacted or involved in a blocking reaction. After 24 h of reaction time, very few unreacted resin molecules remain, indicating that curing or post-curing did not significantly complete the resin–hardener reaction. Other molecules such as C_14_H_7_O^+^, CHO^+^, and CH_3_O^+^ follow a similar trend, reducing to nearly undetectable levels after the 24-h reaction period [28,29,30,31].

From this information, we conclude that after 24 h of reaction time, most resin ions, such as C_21_H_24_O_4_^+^, have reacted, leaving only a limited number of unreacted molecules. This suggests that the curing or post-curing process did not significantly enhance the completion of the resin–hardener reaction. The trend observed with other ions like C_14_H_7_O^+^, CHO^+^, and CH_3_O^+^ supports this conclusion.

Regarding the surface tension of C_21_H_24_O_4_, which is 45.6 ± 3 dyne/cm, this value indicates the intermolecular forces at the surface of the resin molecule. Surface tension is a critical property affecting how the resin spreads, wets, and adheres to surfaces. A surface tension of 45.6 ± 3 dyne/cm suggests that C_21_H_24_O_4_ has a moderate level of surface tension, influencing its behavior in applications such as coatings, adhesives, and composites. Specifically, this level of surface tension indicates that the resin can form a relatively stable film and has good wetting properties, essential for creating strong bonds and uniform coatings in various industrial applications.

### 3.2. Remaining Unreacted Hardener

**Figure 4:**
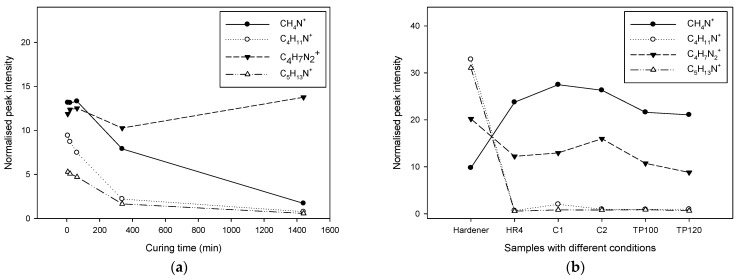
ToF-SIMS normalised peak intensity as a function of (**a**) curing time, and (**b**) samples with different curing conditions for molecules that are associated with the hardener.

The observed behaviour of hardener molecules during the curing and post-curing processes can be explained by the chemical dynamics involved (Figure 4). Initially, the concentration of hardener molecules decreases as they react with the resin. However, during post-curing, particularly at moderate temperatures, some intermediate products or unreacted hardener molecules might become more mobile and migrate to the surface, resulting in an apparent increase in their concentration. When subjected to thermal post-cure, the elevated temperatures further drive the reaction to completion, consuming the remaining hardener molecules and reducing their concentration again.

The normalized intensity of the C_5_H_13_N⁺ ion, a characteristic fragment of the isophoronediamine (IPD) curing agent, exhibited a sharp decrease during the initial 400 min of curing, followed by a more gradual decline up to 1400 min. This trend suggests a two-phase consumption behavior of the amine groups within the epoxy network. In the early stage (0–400 min), the rapid decrease corresponds to the primary cross-linking reaction between the epoxide rings and the reactive amine groups of IPD, resulting in the formation of β-hydroxy ether linkages and rapid incorporation of IPD into the growing polymer matrix. As the reaction progresses, the availability of unreacted epoxy and amine groups diminishes, leading to a slowdown in the reaction kinetics. The slower decline in C_5_H_13_N⁺ intensity beyond 400 min is likely due to the completion of the majority of high-reactivity pathways, with residual amines either trapped within the network or participating in secondary reactions at a reduced rate. Additionally, increased network rigidity during the post-curing phase may further restrict the mobility and surface accessibility of IPD-derived species, contributing to the stabilization of the ion signal at longer curing times.

The C_4_H_7_N_2_^+^ ion, which is tentatively attributed to triazole-based accelerators or structurally related nitrogen-containing heterocycles, displayed a slightly decreasing trend during the first 400 min of curing, followed by an unexpected increase in intensity at 1400 min. The initial decline likely reflects the partial incorporation or interaction of the additive within the epoxy matrix during the early stages of cross-linking, reducing its surface concentration or altering its ionization behavior. However, the subsequent increase in signal at prolonged curing times may be attributed to the thermal-driven redistribution or migration of residual low-molecular-weight species, such as unreacted accelerators or their degradation products, toward the polymer surface during the post-curing phase. Additionally, network relaxation and matrix densification at elevated temperatures can enhance surface exposure of previously buried molecular species, making them more accessible to ionization during ToF-SIMS analysis. This delayed rise in C_4_H_7_N_2_^+^ signal may also indicate the thermal decomposition or rearrangement of cured structures, releasing triazole-containing fragments or related species detectable by secondary ion mass spectrometry.

Isophorone diamine (IPD), a commonly used hardener in epoxy formulations, plays a crucial role in reacting with epoxide groups to form crosslinked networks, significantly influencing the mechanical properties of the cured resin. During the curing process, IPD is expected to be consumed as it reacts with the resin. The observed variations in its concentration during post-curing stages can be attributed to the mobilization of unreacted IPD or its intermediates and the completion of reactions under thermal post-curing conditions.

Understanding these molecular behaviors provides insights into the curing dynamics of epoxy resins. By analyzing the concentration trends of different molecules using techniques like ToF-SIMS, we can optimize curing and post-curing processes to achieve the desired properties in the final product.

### 3.3. Crosslinking Density

**Figure 5:**
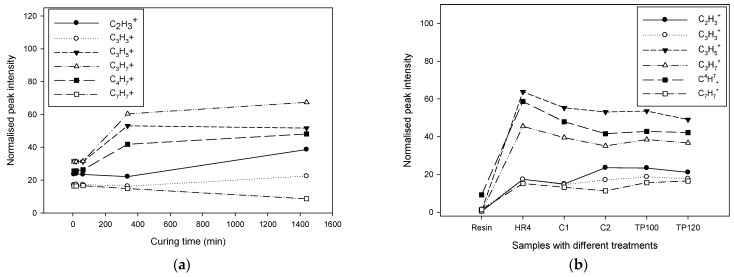
ToF-SIMS normalised peak intensity as a function of (**a**) curing time and (**b**) samples with different curing conditions for molecules that are associated with crosslinking density.

Considering the observations from Figure 5 and their relation to crosslinking density, the ions C_3_H_5_^+^, C_4_H_7_^+^, and C_3_H_7_^+^ exhibit the most notable trends. Initially, the intensity of these molecules increases with the curing reaction up to 24 h, indicating that their concentrations rise as the curing progresses. However, this intensity tends to decrease during further curing and post-curing stages. This decrease in intensity suggests a reduction in their concentrations, which could be associated with changes in cross-linking density.

The observed reduction in the intensity of C_3_H_5_^+^, C_4_H_7_^+^, and C_3_H_7_^+^ after the initial increase may imply that some cross-links are breaking down during the post-curing phase. This hypothesis aligns with the notion that post-curing might lead to a decrease in cross-linking density. It is important to investigate whether thermal post-curing significantly impacts the cross-linking density, as this could influence the final mechanical and chemical properties of the epoxy resin. The declining intensity of C_3_H_5_^+^, C_4_H_7_^+^, and C_3_H_7_^+^ ions during post-curing is attributed to reduced fragmentation due to immobilization within the growing polymer network. An alternative explanation is the decreasing ease of ionization as the surrounding matrix becomes more rigid and less conducive to ion escape. These factors must be considered together when interpreting SIMS data in polymer systems.

In contrast, other ions in the study show a different trend. They exhibit an increase in intensity for the first hour of the reaction but then stabilize, remaining constant regardless of further curing or treatment.

This behavior suggests that these ions may not participate actively in the curing process beyond the initial stage and do not significantly affect cross-linking density or other curing dynamics.

Thus, Figure 5 indicates that, while some molecules contribute to changes in cross-linking density, others remain stable after an initial increase, reflecting differing roles in the curing process. To fully understand these dynamics, further investigation is needed to assess how thermal curing affects cross-linking density and the overall properties of the epoxy resin.

### 3.4. Curing Reaction Path

**Figure 6:**
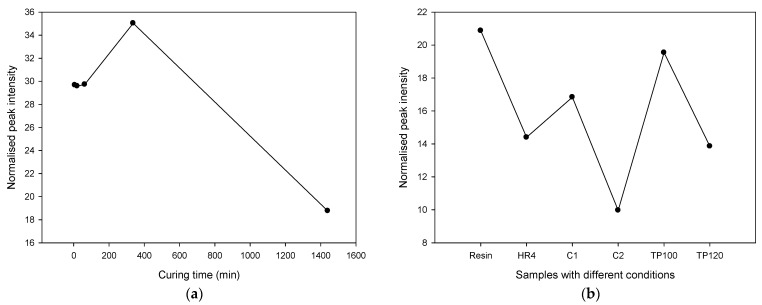
ToF-SIMS normalised peak intensity as a function of (**a**) curing time and (**b**) samples with different curing conditions for the C_7_H_7_O^+^.

Based on the observations provided above and Figure 6, the curing reaction path for the epoxy resin can be described as follows:

*Initial Reaction Phase*—Upon the initiation of the curing process, there is a noticeable increase in the concentration of the C_7_H_7_O molecule. This increase suggests that the curing reaction involves significant levels of blocking, branching, and/or coupling reactions. The rise in C_7_H_7_O^+^ concentration indicates that these reactions are actively occurring as the curing progresses. Specifically, the formation of C_7_H_7_O^+^ might be linked to the creation of intermediate structures or the formation of new linkages between resin and hardener molecules. The C_7_H_7_O^+^ species, commonly associated with the breakdown of the aromatic portion of bisphenol A, was monitored as an indicator of structural changes during curing. Its trend inversely correlates with that of C_14_H_13_O, suggesting its generation is facilitated by the fragmentation of linear oligomer segments.

*Progression Over Time*—As curing continues, the concentration of the C_7_H_7_O^+^ ions initially increases but then begins to decrease. After 24 h of curing, the concentration of C_7_H_7_O^+^ drops below the initial level observed for the resin. This decrease suggests that the molecule is either being consumed or undergoing further reactions that reduce its concentration. The continued reduction in C_7_H_7_O^+^ concentration during the curing phase implies that the intermediate structures or linkages formed earlier are being altered or broken down.

*Post-Curing Effects*—During the post-curing phase, there is a noticeable increase in the concentration of C_7_H_7_O^+^, which rises back to the level of the initial resin concentration. This behavior suggests that post-curing conditions might induce bond breakage or modifications in the network structure. The rise in C_7_H_7_O^+^ concentration during post-curing indicates that some of the bonds formed during the initial curing phase are being broken, leading to an increase in the concentration of this molecule.

*Impact on Cross-Linking Density*—The observed changes in C_7_H_7_O^+^ concentration during post-curing imply that the cross-linking density of the resin network might be affected. The increase in C_7_H_7_O^+^ concentration during post-curing, coupled with its reduction during curing, points towards potential bond breakage or changes in the network structure. This bond breakage could lead to a decrease in cross-linking density, which is critical for the final mechanical and chemical properties of the epoxy resin.

The observed trend of the C_7_H_7_O^+^ ion signal correlates with the progression of the curing process. This behavior suggests that C_7_H_7_O^+^ can serve as a qualitative indicator of early-stage network formation. The initial rise likely reflects the generation of phenolic or aromatic ether structures during the crosslinking reactions, while the subsequent evolution implies that the majority of reactive sites have been consumed as the network approaches completion. This supports its potential role as a marker of crosslink density evolution during epoxy curing. Similar trends have been reported in previous studies utilizing similar techniques to monitor chemical transformations during thermoset curing [23,24].

The curing reaction path, as depicted by the behavior of C_7_H_7_O^+^, illustrates a complex interplay of blocking, branching, and coupling reactions during the initial curing phase, followed by a reduction in the concentration of this molecule over time. The post-curing phase then induces changes that increase the concentration of C_7_H_7_O^+^, likely due to bond breakage or network modifications. This suggests that post-curing conditions might lead to reduced cross-linking density, which could impact the overall performance of the epoxy resin. Understanding these reaction pathways is crucial for optimizing curing and post-curing conditions to achieve the desired properties in the final epoxy resin product.

### 3.5. Difference Between Curing Time and Post Curing (The Effect of Post Curing)

**Figure 7:**
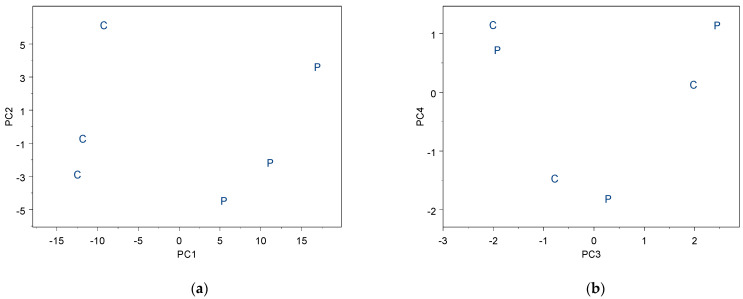
Shows the application of principal components regression analysis (PCR) to capture the relevant information for the curing (noted as C) and post-curing reactions (noted as P) through (**a**) first and second components and (**b**) the third and fourth components after applying. PCR first two components clearly captured the relevant information related to the specific molecular changes that are related to the curing and post-curing reactions.

**Figure 8:**
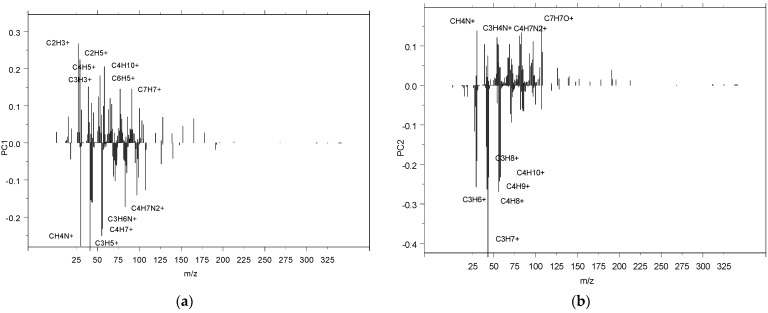
Application of principal components regression analysis (PCR) to capture the loadings (specific molecules) that are most relevant for the curing and post-curing reactions through (**a**) first components and (**b**) the second components. PCR first two components clearly captured the relevant molecules related to the specific changes that are related to the curing and post-curing reactions.

Through the application of Principal Components Regression analysis (PCR), we were able to distinguish the relevant information that enables us to identify the exact molecules that differentiate the cured samples from the post-cured samples. The first two components captured more than 80% of the relevant data and are represented in Figure 7. Furthermore, Figure 8 shows the corresponding loadings for these components and highlights the relevant ions that contribute to understanding the differences between the curing and post-curing processes. The following ions (concentrations) were clearly relevant for distinguishing between the curing and post-curing stages: C_2_H_3_^+^, CH_4_N^+^, C_4_H_5_^+^, C_3_H_5_^+^, C_2_H_5_^+^, and C_4_H_10_^+^ in PC1, and C_3_H_7_^+^, C_3_H_6_^+^, C_4_H_8_^+^, C_4_H_9_^+^, and CH_4_N^+^ in PC2.

Curing time is the period required for an epoxy resin to fully cure at a specific temperature after mixing with the hardener. During this time, the resin and hardener molecules react, forming covalent bonds and cross-linking the polymer chains to create a rigid and thermally stable network [32]. The curing time depends on the type and concentration of the hardener, the temperature, and the curing conditions [33,34,35].

Post-curing is an additional heat treatment applied to the epoxy resin after it has reached its full strength. This process, typically conducted at a higher temperature for a longer duration and in the absence of oxygen, enhances the thermal stability, mechanical properties, and chemical resistance of the cured epoxy resin. Post-curing promotes further cross-linking and the formation of a more extensive covalent network, decreasing residual unreacted functional groups and increasing the degree of cross-linking. This leads to improved mechanical properties, such as stiffness, strength, and toughness.

From ToF-SIMS data, specific molecular changes can be deduced to distinguish between curing and post-curing reactions. During curing, the primary changes involve the initial formation of covalent bonds between resin and hardener molecules. The relevant ions detected, such as C_2_H_3_^+^, CH_4_N^+^, C_4_H_5_^+^, C_3_H_5_^+^, C_2_H_5_^+^, and C_4_H_10_^+^, are indicative of the initial cross-linking process.

In contrast, post-curing results in additional cross-linking and the elimination of residual unreacted groups. The presence of ions such as C_3_H_7_^+^, C_3_H_6_^+^, C_4_H_8_^+^, C_4_H_9_^+^, and CH_4_N^+^ in the post-cured samples indicates a more extensive network of covalent bonds and a higher degree of cross-linking. These molecular changes, observed through the differences in ion concentrations and types, reflect the enhanced mechanical and thermal properties achieved through post-curing.

## 4. Conclusions

Through the application of Principal Components Regression analysis (PCR) on the same resin-to-hardener ratio, we were able to distinguish the molecular differences between cured and post-cured epoxy resin samples. The study focused on variables such as the cross-linking reaction progress, the nature of the cross-links, and the structure of the cured resin. The completion of the curing reaction was associated with specific ions, including C_14_H_7_O^+^, CHO^+^, CH_3_O^+^, and C_21_H_24_O_4_^+^, while the presence of remaining unreacted hardener was indicated by C_21_H_24_O_4_^+^ ions.

The analysis revealed that the cross-linking density and the intensities of aliphatic hydrocarbons played a crucial role in distinguishing between different stages of curing. Calibration was performed to ensure that all intensities totaled to one, and certain ions were tracked to differentiate between uncured, cured, post-cured, and thermally post-cured states. The curing reaction path, branching, and network density were primarily indicated by aliphatic hydrocarbons and C_7_H_7_O^+^ ions.

Negative spectra provided insights into the extent of hardener molecule consumption during curing and post-curing. The study demonstrated that post-curing, involving additional heat treatment, resulted in further cross-linking, the elimination of residual unreacted groups, and the formation of a more extensive network of covalent bonds. This was evidenced by the increased intensity of relevant ions, indicating a higher degree of cross-linking and improved mechanical and thermal properties.

These findings confirm that ToF-SIMS data effectively differentiate curing from post-curing reactions based on molecular changes. The completion of the curing reaction, cross-linking density, and surface energy are crucial factors in understanding the properties of the cured epoxy resin. Post-curing improves these properties by enhancing cross-linking and removing residual unreacted components, yielding a stronger, more thermally stable material. These insights contribute to a better understanding of the curing process and the optimization of epoxy resin properties for various applications.

## Data Availability

The original contributions presented in this study are included in the article. Further inquiries can be directed to the corresponding author.
